# Fermented *Perilla frutescens* Ameliorates Depression-like Behavior in Sleep-Deprivation-Induced Stress Model

**DOI:** 10.3390/ijms24010622

**Published:** 2022-12-30

**Authors:** Hye Jin Jee, Dajung Ryu, Suyeon Kim, Sung Hum Yeon, Rak Ho Son, Seung Hwan Hwang, Yi-Sook Jung

**Affiliations:** 1 College of Pharmacy, Ajou University, Suwon 16499, Republic of Korea; 2 AI-Super Convergence KIURI Translational Research Center, School of Medicine, Ajou University, Suwon 16499, Republic of Korea; 3 Research Institute of Pharmaceutical Sciences and Technology, Ajou University, Suwon 16499, Republic of Korea; 4 R&D Center, Huons Co., Ltd., 55 Hanyangdaehak-ro, Ansan 15588, Republic of Korea

**Keywords:** fermented *Perilla frutescens*, sleep deprivation, stress, depression, corticosterone, BDNF

## Abstract

Excessive stress plays a critical role in the pathogenesis of mood disorders such as depression. Fermented natural products have recently attracted attention because of their health benefits. We evaluated the antidepressant-like efficacy of fermented *Perilla frutescens* (FPF), and its underlying mechanisms, in sleep deprivation (SD)-induced stress mice. SD-stressed mice revealed a remarkable increase in the immobility time in both forced swimming test and tail suspension test; this increase was ameliorated by treatment with FPF at doses of 100 and 150 mg/kg. FPF treatment also reduced the level of stress hormones such as corticosterone and adrenocorticotropic hormone. Additionally, FPF increased the levels of serotonin and dopamine which were significantly decreased in the brain tissues of SD-stressed mice. The increased expression of proinflammatory cytokines, such as TNF-α and IL1β, and the decreased expression of brain-derived neurotrophic factor (BDNF) in the stressed mice were significantly reversed by FPF treatment. Furthermore, FPF also increased phosphorylation of tropomyosin receptor kinase B (TrkB), extracellular regulated protein kinase (ERK), and cAMP response element binding protein (CREB). Among the six components isolated from FPF, protocatechuic acid and luteolin-7-*O*-glucuronide exhibited significant antidepressant-like effects, suggesting that they are major active components. These findings suggest that FPF has therapeutic potential for SD-induced stress, by correcting dysfunction of hypothalamic-pituitary-adrenal axis and modulating the BDNF/TrkB/ERK/CREB signaling pathway.

## 1. Introduction

Stress represents a series of biological responses, including neurological, endocrine, and immunological responses, to strong external stimuli [1]. Excessive stress has been recognized as a risk factor for many diseases, including cardiovascular and metabolic diseases, and neurologic disorders such as depression [2]. Stress-induced depression, which is highly relevant in a modern society constantly and unavoidably exposed to stressors [3], not only poses a serious threat to human health due to its high incidence and mortality rates, but also causes a massive economic burden globally [4,5]. Depressed people experience two and a half times more stressors than non-depressed people, and depression is preceded by a stressful event in 80% of the cases [6]. Stressful experiences have been linked to the onset of major depressive episodes [7,8,9,10]. Although the pathological mechanisms of stress-induced depression are not fully understood, hypothalamic-pituitary-adrenal (HPA) axis hyperactivity is known to participate in the pathogenesis of depression [11,12,13,14]. The HPA axis is a neuroendocrine system which plays an essential role in the production of the stress hormone cortisol: in response to stress, the hypothalamus releases corticotropin-releasing hormone, which induces adrenocorticotropic hormone (ACTH) production in the pituitary gland and peripheral circulation, causing the secretion of plasma glucocorticoids (cortisol in humans, and corticosterone in rodents) from the adrenal cortex [15]. High cortisol concentrations not only induce behavioral changes such as depression and anxiety, but also cause pathological damage to hippocampal neurons [15,16], reducing the production of neurogenic factors such as brain-derived nerve growth factor (BDNF) [14]. In patients with depression, elevated blood cortisol [17] and reduced BDNF expression [18] were accompanied by HPA axis abnormalities [19]. BDNF has been known to exert antidepressant effects by activating tropomyosin-related kinase B (TrkB) [20,21,22].

Sleep, a physiological process of the human body, is important in maintaining homeostasis, and inadequate sleep is detrimental to human health [23,24]. Sleep deprivation (SD) is an important driver of deterioration in neural and other physiological functions [25,26]. SD-induced stress induces transient activation of the neuroendocrine stress system and HPA axis [27]. SD-stressed rodents exhibit elevated plasma stress-hormone levels and expression of inflammatory factors, such as interleukin (IL)-1β and tumor necrosis factor (TNF)-α [28,29]. SD-induced stress is therefore considered a suitable model for inducing depression-like behavior. 

Because of the limitations of existing antidepressants, including adverse effects and difficulties in long-term use, natural products with greater safety and efficacy are attracting attention as alternatives to antidepressants [30,31]. *Perilla frutescens* (Lamiaceae) (PF), an annual herbaceous plant, is used as a traditional medicine and functional food, with edible leaves and seeds [32,33]. PF has antioxidant, anti-inflammatory [34,35], antimicrobial [36], and antidepressant-like effects [37,38]. Nonetheless, its underlying mechanisms remain to be elucidated. 

There is an increasing trend towards minimally processed foods that positively affect physical and mental health [39], with growing interest in foods fermented by microorganisms and enzymatic conversion, which improves their nutrition, safety, and therapeutic efficacy [40,41]. Here, we evaluated the effects of *Bacillus subtilis*-fermented PF (FPF) on depression-like behavior in an SD-induced stress mouse model. We further investigated the underlying mechanisms in vitro as well as in vivo, focusing on the HPA axis and BDNF signaling pathways.

## 2. Results

### 2.1. Effects of FPF on Depression-like Behavior in Sleep-Deprived Mice

**Figure 1:**
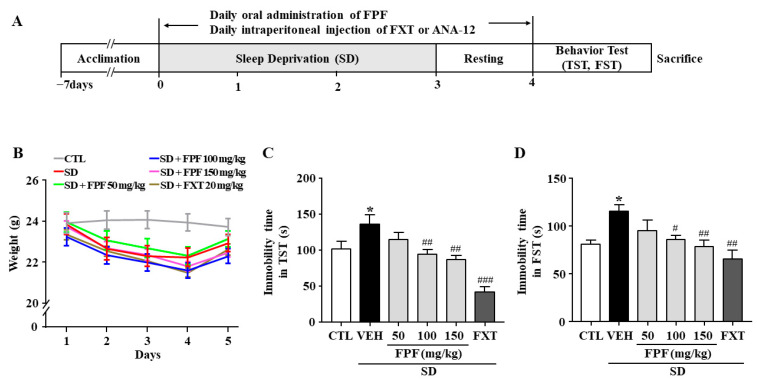
Effects of FPF on SD stress-induced depression-like behavior in mice. (**A**) Scheme for experimental procedures. (**B**) Body weight (two-way ANOVA: Interaction: F_(20, 210)_ = 0.5886, *p* = 0.9182; Days: F_(4, 210)_ = 10.89, *p* < 0.0001; Group: F_(5, 210)_ = 11.52, *p* < 0.0001). (**C**,**D**) Immobility times in the TST (**C**) and FST (**D**). TST (one-way ANOVA: F_(5, 61)_ = 11.59, *p* < 0.0001); FST (one-way ANOVA: F_(5, 57)_ = 4.537, *p* = 0.0015). Mice were exposed to SD-induced stress for 72 h. Mice were treated with FPF (50, 100, 150 mg/kg) by oral administration or FXT (20 mg/kg) by intraperitoneal injection once daily for 5 days. Values represent the mean ± standard error of the mean (*n* = 10). * *p* < 0.05 compared with the control group. # *p* < 0.05, ## *p* < 0.01, and ### *p* < 0.005 compared with the vehicle group. TST: tail suspension test, FST: forced swimming test, CTL: control, VEH: vehicle, FXT: fluoxetine, FPF: fermented *Perilla frutescens*, SD: sleep deprivation.

We assessed the effects of FPF on stress-induced behavior in sleep-deprived mice. As a positive control, we used fluoxetine (FXT), a selective serotonin reuptake inhibitor (SSRI) antidepressant. Body weight was significantly reduced in sleep-deprived mice, and the reduction in body weight was not affected by FPF (50, 100, 150 mg/kg) or FXT (20 mg/kg) treatment (Figure 1B). Relative to the controls, sleep-deprived mice had longer immobility time in the tail suspension test (TST) (136.3 ± 8.75 s, *p* < 0.0001) and forced swimming test (FST) (113.4 ± 10.28 s, *p* < 0.05). FPF treatment significantly reduced their immobility time at 100 mg/kg (TST: 93.38 ± 8.58 s, *p* < 0.01; FST: 86.73 ± 10.28 s, *p* < 0.05) and at 150 mg/kg (TST: 89.38 ± 8.58 s, *p* < 0.001; FST: 78.77 ± 10.54 s, *p* < 0.01), in both the TST and FST (Figure 1C,D). FXT (20 mg/kg) treatment also significantly reduced the immobility time in both the TST (41.45 ± 8.95 s, *p* < 0.0001) and FST (65.91 ± 9.88 s, *p* < 0.001).

### 2.2. Effects of FPF on ACTH and Corticosterone Levels

**Figure 2:**
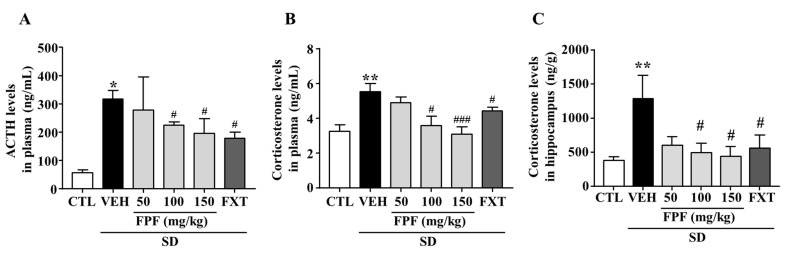
Effects of FPF on plasma levels of ACTH and corticosterone, and hippocampal corticosterone in sleep-deprived mice. (**A**) ACTH (one-way ANOVA: F_(5, 25)_ = 3.58, *p* = 0.0141); (**B**) corticosterone (one-way ANOVA: F_(5, 46)_ = 5.422, *p* = 0.0005); and (**C**) hippocampal corticosterone (one-way ANOVA: F_(5, 30)_ = 3.136, *p* = 0.0215), determined via ELISA. Values represent the mean ± standard error of the mean (*n* = 6). * *p* < 0.05 and ** *p* < 0.01 compared with the control group. # *p* < 0.05 and ### *p* < 0.005 compared with the vehicle group. ACTH: adrenocorticotropic hormone.

To determine whether FPF treatment affects levels of stress-related hormones, we measured changes in the levels of plasma ACTH and corticosterone, and hippocampal corticosterone in sleep-deprived mice. ACTH levels were significantly increased by SD stress (317.9 ± 30.25 ng/mL, *p* < 0.05); this increase was suppressed by treatment with FPF at 100 or 150 mg/kg FPF (224.8 ± 11.18 and 196.1 ± 51.61 ng/mL, respectively; *p* < 0.05), and 20 mg/kg FXT (178.1 ± 22.36 ng/mL, *p* < 0.01) (Figure 2A). Plasma corticosterone levels were significantly increased by SD stress (5.531 ± 0.63 ng/mL, *p* < 0.01); this increase was ameliorated by treatment with 100 or 150 mg/kg FPF (3.587 ± 0.64 ng/mL, *p* < 0.05, and 3.093 ng/mL, *p* < 0.001), and 20 mg/kg FXT (4.42 ± 0.73 ng/mL, *p* < 0.05) (Figure 2B). Hippocampal corticosterone levels were also significantly increased by SD stress (1288 ± 340.6 ng/g, *p* < 0.01); this increase was suppressed by treatment with 100 or 150 mg/kg FPF (495.1 ± 139.5 and 440.1 ± 142.7 ng/g, respectively; *p* < 0.05), and FXT (557.1 ± 195.7 ng/g, *p* < 0.05) (Figure 2C). 

### 2.3. Effects of FPF on Serotonin and Dopamine Concentrations

**Figure 3:**
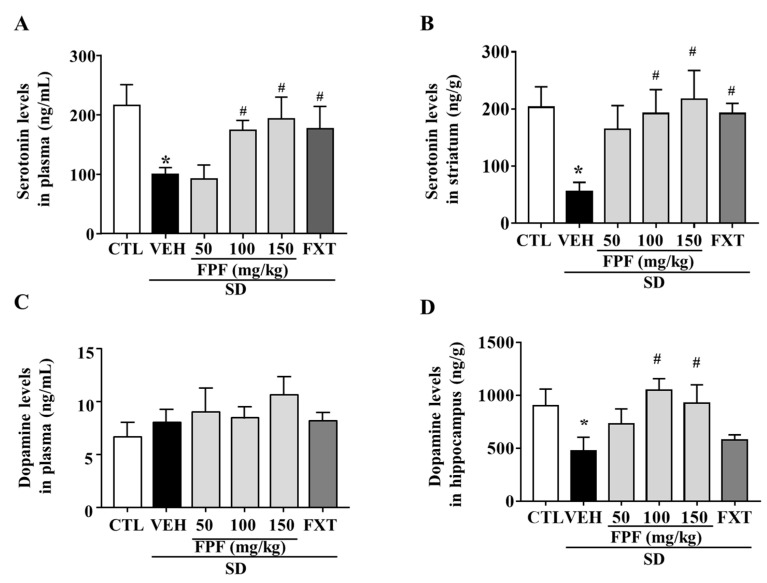
Effects of FPF on serotonin and dopamine levels in the plasma and selected brain regions of sleep-deprived mice. (**A**) Plasma serotonin (one-way ANOVA: F_(5, 18)_ = 3.11, *p* = 0.0339); (**B**) striatal serotonin (one-way ANOVA: F_(5, 30)_ = 2.656, *p* = 0.042); (**C**) plasma dopamine (one-way ANOVA: F_(5, 17)_ = 1.208, *p* = 0.3473) and (**D**) hippocampal dopamine (one way ANOVA: F_(5, 30)_ = 2.787, *p* = 0.035) determined via ELISA. Values represent the mean ± standard error of the mean (*n* = 6). * *p* < 0.05 compared with the control group. # *p* < 0.05 compared with the vehicle group.

To determine whether FPF treatment affected the levels of depression-related neurotransmitters, we measured changes in serotonin and dopamine levels in the plasma and selected brain regions of sleep-deprived mice. Many studies related to stress has focused on the striatum and hippocampus as pivotal brain regions for the stress response, and reports reduced levels of serotonin and dopamine [42,43]. Indeed, we examined the levels of serotonin and dopamine in both hippocampus and striatum in our SD model, and we could observe a remarkable decrease in serotonin level in the striatum, and a remarkable decrease in dopamine level in the hippocampus. Then, we assessed the effect of FPF on serotonin and dopamine levels in those barin regions. Plasma serotonin levels were reduced by SD stress (99.74 ± 11.42 ng/mL, *p* < 0.05); this reduction was suppressed by treatment with FPF at 100 or 150 mg/kg (174 ± 16.62 and 193.2 ± 36.57 ng/mL, respectively; *p* < 0.05), and 20 mg/kg FXT (176.3 ± 37.28 ng/mL, *p* < 0.05) (Figure 3A). Striatal serotonin levels were reduced by SD stress (55.08 ± 16.64 ng/g, *p* < 0.05); this reduction was ameliorated by treatment with FPF at 100 or 150 mg/kg (192 ± 41.89 and 217.3 ± 50.22 ng/g, respectively; *p* < 0.05), and 20 mg/kg FXT (192.2 ± 17.81 ng/g, *p* < 0.05) (Figure 3B). Plasma dopamine levels were not significantly changed by SD stress and FPF treatment (Figure 3C). Hippocampal dopamine levels were reduced by SD stress (475.2 ± 130.1 ng/g, *p* < 0.05); this reduction was suppressed by treatment with FPF at 100 or 150 mg/kg (1052 ± 105.5 and 928 ± 172.2 ng/g, respectively; *p* < 0.05) (Figure 3D).

### 2.4. Effects of FPF on Hippocampal Pro-Inflammatory Cytokine Expression in Sleep-Deprived Mice

**Figure 4:**
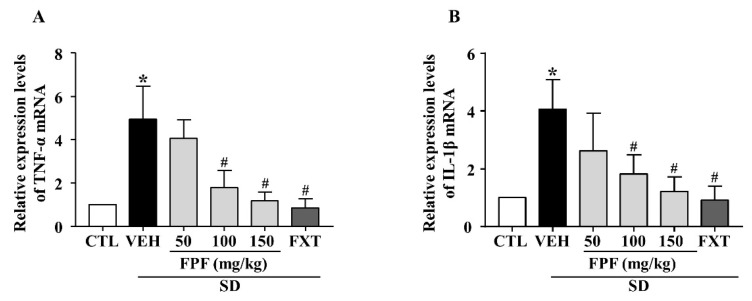
Effects of FPF on hippocampal pro-inflammatory cytokine expression in sleep-deprived mice. (**A**) qRT-PCR of TNF-α mRNA (one-way ANOVA: F_(5, 18)_ = 5.043, *p* = 0.0046), (**B**) qRT-PCR of IL-1β mRNA (one-way ANOVA: F_(5, 32)_ = 3.052, *p* = 0.0231). Values represent the mean ± standard error of the mean (*n* = 6). * *p* < 0.05 compared with the control group. # *p* < 0.05 compared with the vehicle group. IL: interleukin, TNF: tumor necrosis factor.

To investigate the effects of FPF on SD stress-induced inflammatory responses, we examined the mRNA expression level of pro-inflammatory cytokines (TNF-α and IL-1β) using quantitative real time-PCR (qRT-PCR). Their mRNA expression levels were elevated in sleep-deprived mice; these increased levels were significantly decreased by treatment with 100 or 150 mg/kg FPF and 20 mg/kg FXT (Figure 4A,B). 

### 2.5. FPF Increases Hippocampal BDNF Expression in Sleep-Deprived Mice

**Figure 5:**
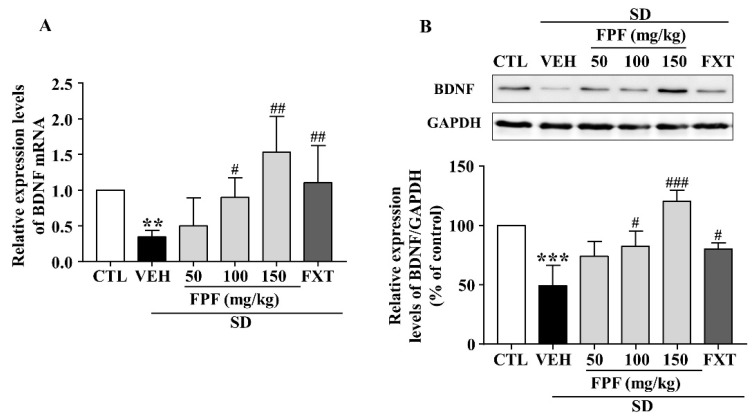
Effects of FPF on hippocampal BDNF expression in sleep-deprived mice. (**A**) qRT-PCR of BDNF mRNA (one-way ANOVA: F_(5, 29)_ = 3.036, *p* = 0.0253). Values represent the mean ± standard error of the mean (*n* = 6). (**B**) Western blot of BDNF. (Upper) Representative Western blot image; (Lower) BDNF expression relative to that in the GAPDH control (one-way ANOVA: F_(5, 12)_ = 14.37, *p* = 0.0001). Values represent the mean ± standard error of the mean (*n* = 3). ** *p* < 0.01 and *** *p* < 0.005 compared with the control group. # *p* < 0.05, ## *p* < 0.01 and ### *p* < 0.005 compared with the vehicle group. BDNF: brain-derived nerve growth factor, GAPDH: glyceraldehyde 3-phosphate dehydrogenase.

To identify the molecular mechanisms underlying the antidepressant-like effect of FPF, we determined BDNF mRNA and protein expression via qRT-PCR and Western blotting. Hippocampal BDNF mRNA levels were significantly decreased in sleep-deprived mice; this reduction was attenuated by treatment with FPF at 100 and 150 mg/kg or FXT at 20 mg/kg (Figure 5A). BDNF protein expression, which was reduced by SD stress, was significantly increased by 100 and 150 mg/kg FPF and 20 mg/kg FXT (Figure 5B).

### 2.6. FPF Activates Hippocampal BDNF/TrkB/ERK/CREB Signaling in Sleep-Deprived Mice

**Figure 6:**
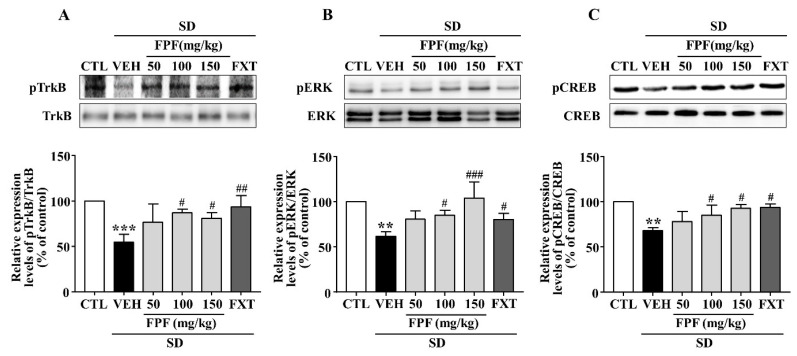
Effects of FPF on hippocampal BDNF/TrkB/ERK/CREB signaling molecules in sleep-deprived mice. Expression of (**A**) pTrkB/TrkB (one-way ANOVA: F_(5, 12)_ = 6.679, *p* = 0.0034), (**B**) pERK/ERK (one-way ANOVA: F_(5, 12)_ = 8.223, *p* = 0.0014), and (**C**) pCREB/CREB (one-way ANOVA: F_(5, 12)_ = 8.477, *p* = 0.0012), determined via Western blot. (Upper) Representative Western blot image; (Lower) expression relative to that in the control. Values represent the mean ± standard error of the mean (*n* = 3). ** *p* < 0.01 and *** *p* < 0.005 compared with the control group. # *p* < 0.05, ## *p* < 0.01 and ### *p* < 0.005 compared with the vehicle group. pTrkB: phosphorylation of tropomyosin receptor kinase B, pERK: phosphorylation of extracellular regulated protein kinase, and pCREB: phosphorylation of cAMP response element binding protein.

Depression is known to be associated with the BDNF/TrkB/ERK/CREB signaling pathway [44,45,46,47]. We therefore examined whether FPF treatment affects BDNF signaling activity, via Western blotting (Figure 6). SD stress resulted in reduced BDNF, p-TrkB, p-ERK and p-CREB expression; these reduced levels were significantly increased by 100 and 150 mg/kg FPF and 20 mg/kg FXT. 

### 2.7. Effects of the TrkB Antagonist ANA-12 on the Antidepressant-like Effect of FPF in Sleep-Deprived Mice

**Figure 7:**
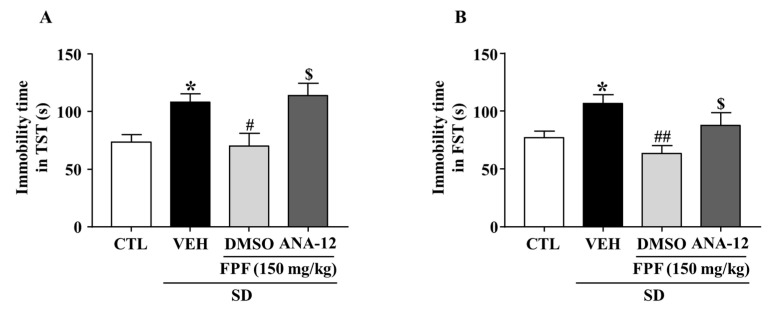
Effects of ANA-12, a TrkB antagonist, on the antidepressant-like effect of FPF in sleep-deprived mice. (**A**,**B**) Comparison of immobility times in (**A**) TST (one-way ANOVA: F_(3, 28)_ = 6.246, *p* = 0.0022) and (**B**) FST (one-way ANOVA: F_(3, 28)_ = 5.194, *p* = 0.0056). Mice were exposed to SD for 72 h. Mice were treated with FPF (150 mg/kg) by oral administration or ANA-12 (0.5 mg/kg) by intraperitoneal injection once daily for 5 days. Values represent the mean ± standard error of the mean (*n* = 10). * *p* < 0.01 compared with the control group. # *p* < 0.05 and ## *p* < 0.01 compared with the vehicle group. $ *p* < 0.05 compared with the FPF 150 mg/kg group. ANA-12: *N*-[2-[(hexahydro-2-oxo-1*H*-azepin-3-yl)amino]carbonyl]phenyl-benzo[*b*]thiophene-2-carboxamide.

To evaluate the role of BDNF/TrkB in the antidepressant mechanism of FPF, we estimated the involvement of BDNF/TrkB signaling, by using ANA-12, a potent BDNF/TrkB antagonist, followed by the TST and FST. FPF at 150 mg/kg reduced the SD stress-induced increase in the immobility time in both the TST (70.13 ± 10.96 s, *p* < 0.05) and FST (63.38 ± 7.063 s, *p* < 0.01). ANA-12 (at 0.5 mg/kg) significantly reversed the effects of FPF (TST: 114.1 ± 10.63 s, *p* < 0.05; FST: 87.75 ± 11.03 s, *p* < 0.05) (Figure 7).

### 2.8. Effects of the TrkB Antagonist ANA-12 on Hippocampal BDNF, pTrkB, pCREB, and pERK Expression in Sleep-Deprived Mice

**Figure 8:**
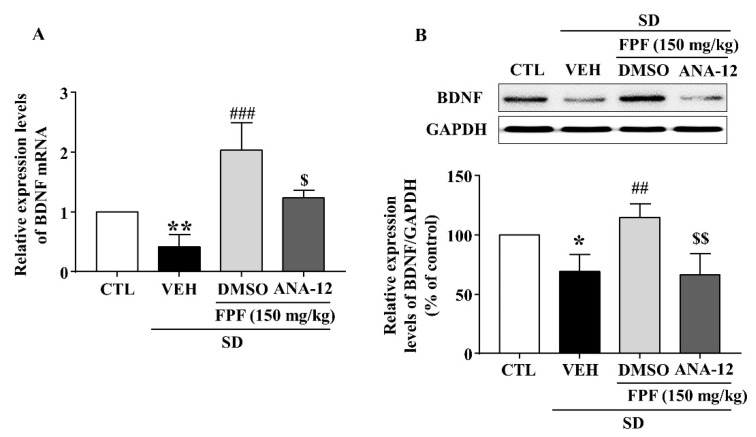
Inhibitory effects of ANA-12 on the FPF-induced increase in hippocampal BDNF expression in sleep-deprived mice. (**A**) qRT-PCR of BDNF mRNA (one-way ANOVA: F_(3, 16)_ = 8.575, *p* = 0.0013). Values represent the mean ± standard error of the mean (*n* = 6). (**B**) Western blot of BDNF (one-way ANOVA: F_(3, 8)_ = 10.18, *p* = 0.0042). (Upper) Representative Western blot; (Lower) BDNF and GAPDH expression. Values represent the mean ± standard error of the mean (*n* = 3). * *p* < 0.05 and ** *p* < 0.01 compared with the control group. ## *p* < 0.01, and ### *p* < 0.005 compared with the vehicle group. $ *p* < 0.05 and $$ *p* < 0.01 compared with the 150 mg/kg FPF group. DMSO: dimethyl sulfoxide.

**Figure 9:**
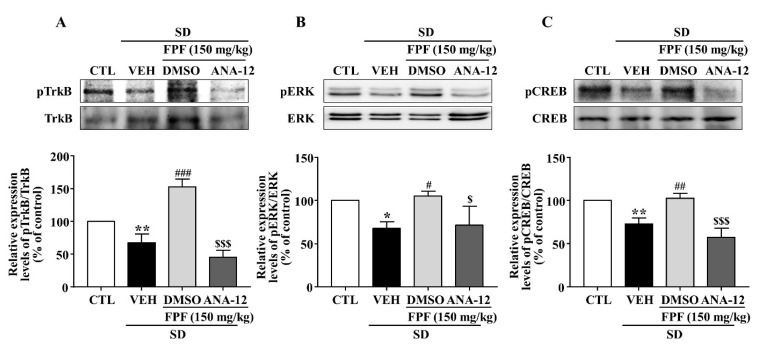
Inhibitory effects of ANA-12 on FPF-induced increases in hippocampal p-TrkB, p-ERK, and p-CREB expression in sleep-deprived mice. Expression of (**A**) pTrkB/TrkB (one-way ANOVA: F_(3, 8)_ = 58.54, *p* < 0.0001), (**B**) pERK/ERK (one-way ANOVA: F_(3, 8)_ = 7.486, *p* = 0.0104), and (**C**) pCREB/CREB (one-way ANOVA: F_(3, 8)_ = 27.4, *p* = 0.0001), determined via Western blotting. (Upper) Representative Western blot; (Lower) relative expression. Values represent the mean ± standard error of the mean (*n* = 3). * *p* < 0.05 and ** *p* < 0.01 compared with the control group. # *p* < 0.05, ## *p* < 0.01 and ### *p* < 0.005 compared with the vehicle group. $ *p* < 0.05 and $$$ *p* < 0.005 compared with the 150 mg/kg FPF group.

In sleep-deprived mice, ANA-12 co-treatment inhibited the increases induced by 150 mg/kg FPF in hippocampal expression of BDNF mRNA and protein (Figure 8) and of pTrkB, pCREB, and pERK (Figure 9). 

### 2.9. Effects of FPF on the Corticosterone-Induced Reduction in SH-SY5Y Cell Viability

**Figure 10:**
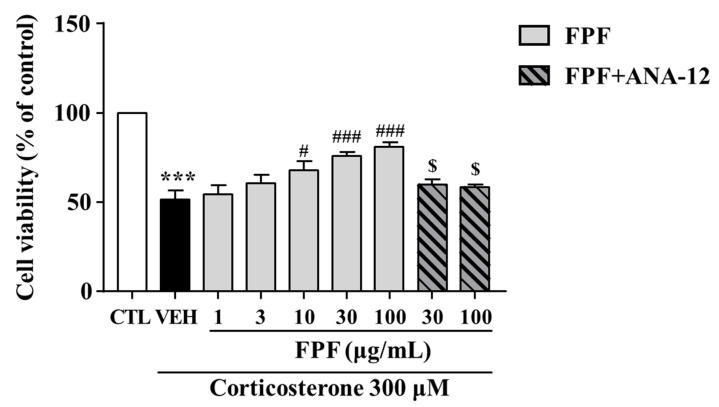
Effects of FPF on cell viability in corticosterone-exposed SH-SY5Y cells. Cells were pretreated with the indicated concentrations of FPF or ANA-12 for 1 h, then exposed to corticosterone for 24 h. Cell viability was measured by MTT assay (one-way ANOVA: F_(8, 56)_ = 16.24, *p* < 0.0001). Values represent the mean ± standard error of the mean (*n* = 6). *** *p* < 0.005 compared with the control group. # *p* < 0.05 and ### *p* < 0.005 compared with the vehicle group. $ *p* < 0.05 compared with the 30 or 100 µg/mL FPF-treated groups.

We investigated the protective effects of FPF against corticosterone-induced reductions in SH-SY5Y cell viability. FPF at 10, 30, and 100 μg/mL prevented the corticosterone-induced reduction in cell viability (at 300 μM corticosterone) in a concentration-dependent manner. ANA-12 co-treatment abolished this protective effect of FPF (Figure 10). 

### 2.10. Effects of FPF and the TrkB Antagonist ANA-12 on BDNF, pTrkB, pCREB, and pERK Expression in Corticosterone-Exposed SH-SY5Y Cells 

**Figure 11:**
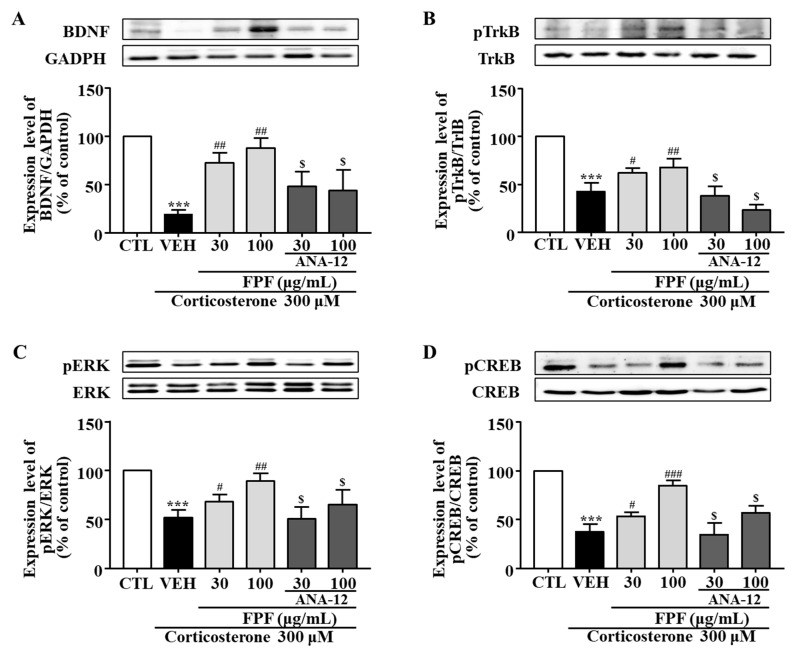
Effects of FPF and ANA-12 on BDNF, p-TrkB, p-ERK, and p-CREB expression in corticosterone-exposed SH-SY5Y cells. (**A**–**D**) Effects of FPF on the expression of (**A**) BDNF/GAPDH (one-way ANOVA: F_(5, 12)_ = 17.66, *p* < 0.0001), (**B**) pTrkB/TrkB (one-way ANOVA: F_(5, 12)_ = 39.85, *p* < 0.0001), (**C**) pERK/ERK (one-way ANOVA: F_(5, 12)_ = 13.47, *p* = 0.0001), and (**D**) pCREB/CREB (one-way ANOVA: F_(5, 12)_ = 41.07, *p* < 0.0001) in corticosterone-stimulated SH-SY5Y cells. (Upper) Representative Western blot; (Lower) relative expression. Values represent the mean ± standard error of the mean (*n* = 3). *** *p* < 0.005 compared with the control group. # *p* < 0.05, ## *p* < 0.01, and ### *p* < 0.005 compared with the vehicle group. $ *p* < 0.05 compared with the 30 or 100 µg/mL FPF-treated groups.

In SH-SY5Y cells, BDNF, pTrkB, pCREB, and pERK expression levels were significantly reduced by corticosterone treatment; these reductions were significantly ameliorated by 30 and 100 μg/mL FPF. ANA-12 co-treatment abolished these FPF-induced increases (Figure 11). 

### 2.11. Effects of FPF Compounds on the Corticosterone-Induced Reduction in SH-SY5Y Cell Viability

**Figure 12:**
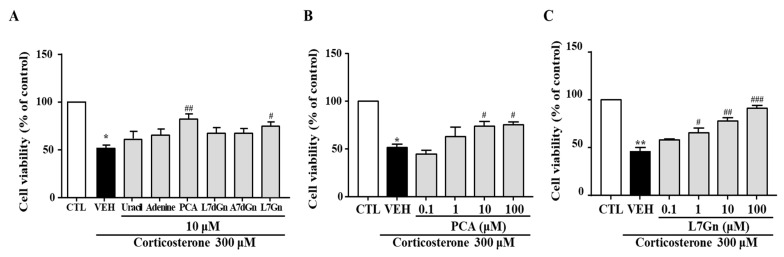
Effects of FPF compounds on corticosterone-exposed SH-SY5Y cell viability. (**A**) Effects of six FPF compounds (10 μM) (one-way ANOVA: F_(7, 40)_ = 16.64, *p* < 0.0001). Concentration-dependent effects of (**B**) PCA (one-way ANOVA: F_(5, 15)_ = 15.83, *p* < 0.0001) and (**C**) L7Gn (one-way ANOVA: F_(5, 26)_ = 33.13, *p* < 0.0001). Cells were treated with each compound for 24 h; viability was measured via MTT assay. Values represent the mean ± standard error of the mean (*n* = 6). * *p* < 0.05 and ** *p* < 0.01 compared with the control group. # *p* < 0.05, ## *p* < 0.01 and ### *p* < 0.005 compared with the vehicle group. PCA: protocatechuic acid, L7dGn: luteolin-7-*O*-diglucuronide, A7dGn: apigenin-7-*O*-diglucuronide, L7Gn: luteolin-7-*O*-glucuronide.

To further clarify which components of FPF participate in its antidepressant-like activity, six compounds (uracil, adenine, PCA, L7dGn, A7dGn, and L7Gn) isolated from FPF were evaluated using an in vitro stress model. The corticosterone-induced reductions in cell viability were significantly reversed by PCA and L7Gn (Figure 12A). These neuroprotective effects were concentration-dependent (Figure 12B,C).

### 2.12. Effects of PCA and L7Gn on SD-Stress Induced Depression-like Behavior

**Figure 13:**
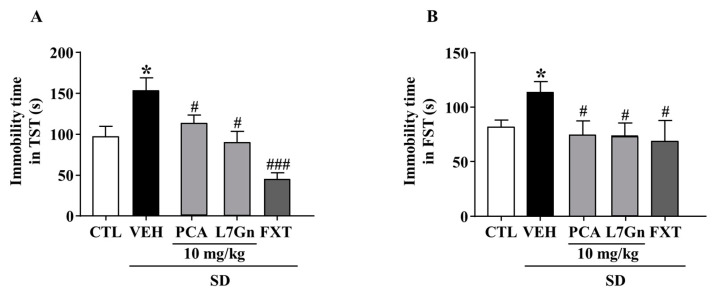
Effects of PCA and L7Gn on SD stress-induced depression-like behavior. (**A**,**B**) Immobility time in (**A**) the TST (one-way ANOVA: F_(5, 26)_ = 7.471, *p* = 0.0002) and (**B**) the FST (one-way ANOVA: F_(5,25)_ = 3.084, *p* = 0.04). Mice were exposed to SD stress for 72 h. Mice were treated with PCA and L7Gn (10 mg/kg) via oral administration or FXT (20 mg/kg) via intraperitoneal injection once daily for 5 days. Values represent the mean ± standard error of the mean (*n* = 8). * *p* < 0.05 compared with the control group. # *p* < 0.05 and ### *p* < 0.005 compared with the vehicle group.

We further evaluated the effects of FPF components on SD-stress induced depression-like behavior. The SD-stress induced elevated immobility times were significantly (*p* < 0.05) attenuated by treatment with PCA (TST: 112.2 ± 15.54 s; FST: 74.6 ± 15.96 s) and L7Gn (TST: 88.83 ± 18.37 s; FST: 73.9 ± 9.88 s) (Figure 13A,B). 

## 3. Discussion

There is increasing interest in natural products, and in particular fermented foods, as alternatives to conventional medication. Here, in a mouse model of depression, FPF ameliorated SD stress-induced depression-like behavior, reducing SD-stress-elevated plasma ACTH and corticosterone levels and TNF-α and IL-1β mRNA expression. FPF treatment rescued the SD-stress-induced reductions in BDNF expression and TrkB, ERK, and CREB phosphorylation, and its effects were reversed by a TrkB antagonist. These results suggest that FPF has antidepressant-like efficacy, possibly by modulating the HPA axis and the BDNF/TrkB/ERK/CREB signaling pathway.

Depression has numerous causes, including psychological stress and biological derangement, and stress plays a pivotal role in its development. Although the pathologic mechanisms of stress-induced depression are not yet thoroughly understood, HPA axis dysfunction has been suggested as a risk factor, given that most depressed patients exhibit hypersecretion of cortisol [48] and impairment of the cortisol negative-feedback system [49]. Further, exposure to high levels of cortisol has been reported to cause brain damage, especially in the hippocampus or cortex, in which glucocorticoid receptors are abundant [50]. Hippocampal volume is smaller in depressed patients than in healthy controls [51]. Moreover, glucocorticoid overexposure has been implicated in hippocampal apoptosis and depression-like behaviors in depressed patients and rodents [52,53]. 

The association between HPA axis dysfunction and subsequent depression has been demonstrated in SD-stressed mice [54]. SD stress pathologically activates the HPA axis, causing elevated secretion of corticosterone and downregulated hippocampal neurogenesis, finally resulting in depression [28,55,56]. We therefore used SD-stressed mice as a depression model. Consistent with other studies, our SD-stressed mice exhibited depression-like behavior, including increased immobility time in the forced swimming and tail suspension tests, tests typically used to measure depression-like behavior. However, it has recently been proposed that, rather than measuring depression-like behavior, the FST measures stress-coping mechanisms [57,58], and thus reflects adaptability in the face of stress. Therefore, our finding that FPF (at 100 or 150 mg/kg) reducing the immobility time in both of these tests probably indicates its ameliorating effects on depression-like, as well as stress-coping, behavior. 

Stress-induced activation of the HPA axis has been shown to induce a reduction in monoamine neurotransmitters such as serotonin and dopamine in the central and peripheral nervous system [59,60]. The main brain regions affected by depression are hippocampus, striatum, hypothalamus and prefrontal cortex [61,62]. Additionally, abnormal monoamine levels in these brain regions have been reported to be associated with depressive states, and many antidepressants function by increasing the levels of these monoamines [63,64]. Here, consistent with these studies, we observed that the levels of ACTH and corticosterone are elevated, and the levels of serotonin and dopamine are decreased in SD-stressed mice. All these changes in stress hormones and monoamines were significantly ameliorated by treatment with FPF at 100 and 150 mg/kg. These findings indicate that FPF has antidepressant-like efficacy possibly via reducing corticosterone levels and regulating the HPA axis. 

Inflammation and depression are widely known to be associated, with most depressed patients exhibiting elevated inflammation markers such as cytokines (IL-1, IL-6, TNF-α) and hormones (ACTH, glucocorticoid) [65,66]. Inflammatory responses can affect neurotransmitter synthesis and glucocorticoid resistance, and the resulting neurodegeneration can contribute to depression [67]. SD stress leads to increases in the levels of pro-inflammatory cytokines such as IL-1β, IL-6, and TNF-α [68,69]. Consistent with these reports, we found that SD stress increased hippocampal IL-1β and TNFα mRNA expression, whereas FPF treatment significantly rescued these levels (Figure 4A,B).

BDNF, a critical neurotrophin in the etiology of depression [70,71] and essential signaling molecule in nervous system development, is responsible for brain neuronal survival, synapse formation, and synaptic plasticity [72]. It has therefore been a target in neuropsychiatric treatment, including for mood disorders [44]. BDNF expression is reduced in the brains of depressed animals and human patients [73,74]. Here, hippocampal mRNA and protein expression of BDNF was lower in SD-stressed mice than in control mice, and FPF treatment remarkably rescued BDNF expression (Figure 5). BDNF binds preferentially to the TrkB receptor, and BDNF/TrkB system dysfunction correlates with pathophysiology in psychiatric disorders [75,76]. After binding to TrkB, BDNF activates the MAPK cascade, of which the ERK pathway is one of the best characterized signaling pathways [77,78]. ERK1/2 proteins are known to play important roles in regulating cell survival, proliferation, and differentiation [79,80]. Depressed and suicidal patients exhibit significantly lower ERK expression in the prefrontal cortex [81]. Further, p-ERK is associated with depressive symptoms, and antidepressants can alleviate depression-like behavior by increasing p-ERK expression [82,83]. CREB, which can be activated by ERK, must be converted to p-CREB to achieve transcriptional activity and regulatory function [82]. Postmortem and clinical studies have revealed significantly lower CREB expression in patients with depression than in normal controls [84]. Chronically stressed mice exhibit reduced hippocampal and frontal CREB levels [85,86]. Here, FPF treatment rescued the SD-stress-induced reductions in TrkB, ERK, and CREB phosphorylation (Figure 6). ANA-12, a potent TrkB antagonist, abolished the antidepressant-like effects (Figure 7), and blocked the enhancing effects of 150 mg/kg FPF on BDNF, p-TrkB, p-ERK, and p-CREB expression (Figure 8 and Figure 9). These findings suggest that the antidepressant-like effect of FPF may involve upregulation of hippocampal BDNF/TrkB/ERK/CREB signaling. Further study is needed to clarify the exact mechanisms by which FPF upregulates BDNF mRNA expression.

Most patients with depression exhibit cortisol hypersecretion, with resultant brain tissue damage. Corticosterone exposure are known to induce neuronal cell death by reducing hippocampal BDNF expression [87]. Based on this, glucocorticoid-induced neuronal cell injury has been widely used as an in vitro model of depression [88,89]. Therefore, we further examined the effects of FPF and its underlying mechanisms, in an in vitro model of depression that mimics glucocorticoid hypersecretion by exposing SH-SY5Y cells to corticosterone. Consistent with other studies, our study showed that corticosterone exposure for 24 h significantly reduced cell viability, BDNF expression, and TrkB/ERK/CREB signaling-molecule phosphorylation. FPF reversed all these corticosterone-induced alterations in a concentration-dependent manner (Figure 10 and Figure 11). ANA-12 co-treatment abolished both the neuroprotective effects of FPF and its enhanced phosphorylation of BDNF/TrkB/ERK/CREB molecules, suggesting that FPF can protect neurons from corticosterone-induced cell death by activating the BDNF/Trkb/ERK/CREB signaling pathway.

Since it was confirmed that the in vitro results for FPF were very consistent with the in vivo results, we performed further study to evaluate the efficacy of various components isolated from FPF using corticosterone-exposed SH-SY5Y cell model. Among the six components which were identified during our preliminary investigations (uracil, adenine, PCA, L7dGn, A7dGn, and L7Gn), PCA and L7Gn were shown to elicit significant protective effect on corticosterone-reduced neuronal death (Figure 12). PCA and L7Gn were further found to have ameliorating effect on depression-like and stress-coping behaviors in SD-stressed mice (Figure 13). In addition, the effect of reducing the increase in corticosterone caused by stress was also confirmed (Supplementary Figure S1).

These findings suggest that PCA and L7Gn may be the primary active substances contributing to the antidepressant-like effects of FPF. Consistent with this, we have previously demonstrated that L7Gn improves depression-like behavior by activating BDNF signaling [90].

In conclusion, the results from this study suggest that FPF has therapeutic potential for SD-induced stress, by correcting HPA axis dysfunction and modulating the BDNF/TrkB/ERK/CREB signaling pathway.

## 4. Materials and Methods

### 4.1. Materials

Fermented *Perilla frutescens* (FPF), uracil, adenine, PCA, L7dGn, A7dGn and L7Gn were provided by Huons Co Ltd. (Seoul, Republic of Korea). Corticosterone, FXT and *N*-[2-[(hexahydro-2-oxo-1*H*-azepin-3-yl)amino]carbonyl]phenyl-benzo[*b*]thiophene-2-carboxamide (ANA-12) were purchased from Sigma-Aldrich (Saint Louis, MO, USA). Corticosterone enzyme-linked immunosorbent assay (ELISA) kit and dopamine ELISA kit were purchased from Enzo Life Sciences (Farmingdale, NY, USA). Serotonin ELISA kit and ACTH ELISA kit were purchased from Abcam (Cambridge, UK). Anti-BDNF, anti-CREB, anti-pCREB, anti-ERK, anti-pERK, anti-TrkB, and anti-GAPDH antibodies were purchased from Cell Signaling Technology (Danvers, MA, USA). Anti-pTrkB antibody was purchased from Abcam (Cambridge, MA, USA). All other chemical reagents were purchased from Sigma-Aldrich and were of analytical or HPLC grade.

### 4.2. Extraction and Fermentation of PF

Dried leaves of *Perilla frutescens* (PF) were purchased from Daemyung pharm. Co., Ltd. (Seoul, Republic of Korea). Voucher specimen was deposited at the Institute of Pharmaceutical Technology, Hanyang University, Republic of Korea. The PF (26.0 kg) was extracted in distilled water (650 L × 2 times) under reflux for 5 h and filtered with a 100-mesh filter. The resulting extract was evaporated to afford the extract containing about 20% solid contents (or 20 brix %) in vacuo at 60 °C. The PF extracts (20%, *w*/*v*) were fermented with *Bacillus subtilis* MORI KCCM10450 by incubation 37 °C for 4 days in a medium consisting of lactose (3%, *w*/*v*) and whole milk powder (0.5%, *w*/*v*). *B. subtilis* MORI KCCM10450 were grown in 8 L of 0.6% yeast extract medium and incubated at 37 °C for 23 h as the pre-culture. The fermented broth was then sterilized and evaporated under reduced pressure at 65 °C. Finally, the resulting concentrate was blended with 20 DE malt dextrin and spray dried in a pilot scale spray dryer and yielded 14.69 kg (56.5%).

### 4.3. Isolation of Compounds in FPF

The FPF was suspended (100 g) in distilled water and chromatographed over Diaion HP-20 using gradient solvent system of methanol (MeOH)/water (*v*/*v*) (0:100, 3:7, 6:4, and 100:0) to give seven main fractions (fr.1–fr.7). Fr. 2–4 (10.7 g) were partitioned sequentially with ethyl acetate (EtOAc, 2.15 g), n-butanol (n-BuOH, 2.17 g) and water residue (water, 4.48 g). The n-BuOH fraction (2.17 g) was purified by a Prep HPLC with 0.1% trifluoroacetic acid/MeOH gradient system to yield compounds I (uracil, 6.3 mg) [91] and II (adenine, 3.14 mg) [92]. The EtOAc fraction (2.15 g) was chromatographed over silica gel using isocratic solvent system of chloro-form (CHCl_3_)/MeOH (5:1) to give compound III (PCA, 2.2 mg) [93]. The water fraction (4.48 g) was purified by a Prep HPLC with 0.1% trifluoroacetic ac-id/MeOH gradient system to yield compounds IV (L7dGn, 110.1 mg) and V (A7dGn, 56.6 mg) [94]. The HP-20 Fr. 6 (9.64 g) was chromatographed by Sephadex LH-20 column chromatography using MeOH to obtain compound VI (L7Gn, 43.2 mg) [95]. These compounds were profiled HPLC-UV wavelength at 254 nm, and identified by comparing ^1^H and ^13^C NMR spectra with previously report-ed data. HPLC was performed using an Agilent 1260 system (Agilent, Sunnyvale, CA, USA).

### 4.4. Animals

Male c57BL/6 mice, aged 7 weeks, were purchased from Orient Bio Inc. (Seongnam, Republic of Korea). The mice were housed at 22–24 °C with tap water and food ad libitum. The light/dark cycle of the room was altered every 12 h. The mice were adapted for at least 1 week prior to the experiment. All experimental protocols were conducted with the approval of the Institutional Animal Care and Use Committee (IACUC) of Ajou University (Approval Number 2020-0052).

### 4.5. SD-Induced Stress Model

SD was performed using the modified multiple platform method [96]. Mice were placed in a water tank (42 cm × 26 cm × 18 cm) each containing 8 cylindrical acrylic platforms (3 cm in diameter) (5 cm in distance) and filled with tap water to 1 cm below the platform surface. Each mouse was placed on the platform of a water tank without touching the water. The mice could move within the tank and jump to platforms, but when trying to sleep they fell and could not sleep. During the 72 h SD period, the mice had free access to water and food, and water was changed once a day during the study period.

### 4.6. Experimental Design

Figure 1A displays an overview of the experiment. After 7 days of acclimation, each group was exposed to 72 h SD using a multi-platform method or remained in home cages to act as controls. FPF (50,100 and 150 mg/kg, p.o.) and FXT (20 mg/kg, i.p.) were treated in mice for 5 days including 72 h SD. ANA-12 (0.5 mg/kg, i.p.) was treated 15 min prior to FPF treatment. 5 days after sample treatment, mice underwent depression-related behavioral tests starting 30 min after sample administration according to the protocol, and then sacrificed for Western blot, qRT-PCR and ELISA analyses. The body weight of the mice was measured once daily for five days. All behavioral tests were performed between 10:00 and 17:00.

### 4.7. Tail Suspension Test (TST)

The TST was used to analyze mouse depression-like behavior. Mice were hung with adhesive tape about 1 cm from the tip of their tails and their heads were placed about 50 cm from the floor. The test was performed for 6 min and no movement was defined as immobility. The first 2 min of activity was considered the pre-test period, and immobility time was measured by video recording for the last 4 min.

### 4.8. Forced Swim Test (FST)

The FST was a widely used paradigm for evaluating depression-like behavior [97] or stress coping behavior [57]. Mice were individually forced to swim in an open cylindrical container (14 cm × 19 cm), containing tap water at a temperature of 24–26 °C and a depth of about 13 cm so that they could not escape or touch the bottom. The test was conducted for 6 min, the initial 2 min of activity was the pre-test period, and immobility time was measured by video recording for the last 4 min. Each mouse was judged immobile when floating in the water, making only the minimal movements necessary to keep its head above the water.

### 4.9. Enzyme-Linked Immune-Specific Assay

After behavioral tests were performed, the mice were anesthetized and blood was collected from the abdominal vein. Plasma samples were prepared by centrifugation of the collected blood samples (1000× *g* for 15 min) at 6 °C and then stored at −80 °C until experimentation. The brain tissue samples were weighed and 100–300 μL lysis buffer was added. The samples were homogenized and centrifuged at 4 °C for 20 min. The supernatant was stored at −80 °C until analysis. The concentration of ACTH, corticosterone, serotonin, and dopamine in plasma or brain tissue were analyzed using ACTH ELISA kit (Abcam, Cambridge, UK), corticosterone ELISA Kit (Enzo Life Sciences, Farmingdale, NY, USA), serotonin ELISA Kit (Abcam, Cambridge, UK), and dopamine ELISA kit (Enzo Life Sciences, Farmingdale, NY, USA). After reagents were added according to the manufacturer’s instructions, absorbance was read using a Bio-Tek Synergy HT plate reader (Bio-Tek Instruments Inc., Winooski, VT, USA).

### 4.10. Cell Culture

The human neuroblastoma cell line SH-SY5Y (Korean Cell Line Bank, Seoul, Republic of Korea) was cultured in Dulbecco’s modified Eagle’s medium (Welgene, Gyeongsan, Republic of Korea) supplemented with 10% fetal bovine serum (Gibco, Grand Island, NY, USA), 100 unit/mL penicillin and 100 ug/mL streptomycin solution (Welgene, Gyeongsan, Republic of Korea). Cells were maintained at 37 °C in humidified 95% air and 5% CO_2_ atmosphere.

### 4.11. Cell Viability Assay

SH-SY5Y cells were seeded into 96-well plates (10^5^ cells/well) and left for 24 h prior to being cultured in a serum-free medium for 4 h. Subsequently, the cells were preincubated with or without FPF and ANA-12 for 1 h followed by incubation with 300 μM corticosterone for 24 h. Cells were treated with the 3-(4,5-dimethylthiazol-2-yl)-2,5-diphenyltetrazolium bromide (MTT) reagent (5 mg/mL) for 4 h and the formazan crystals were dissolved by DMSO. After 30 min incubation, cell viability was quantified by measuring the optical density at 570 nm using a microplate reader (Bio-Tek Instruments Inc., Winooski, VT, USA).

### 4.12. Western Blot Analysis

The brain tissue samples and SH-SY5Y cells were lysed and homogenized in cold RIPA buffer (150 mM NaCl, 1% Triton X-100, 0.5% deoxycholic acid, 0.1% sodium dodecyl sulfate, 50 mM Tris-Cl, pH 7.5). The solution was centrifuged at 14,000 rpm for 15 min at 4 °C and the supernatant protein concentration was determined using a commercial BCA assay kit (Thermo Scientific, Waltham, MA, USA). To separate proteins, electrophoresis was conducted, and they were transferred to PVDF membranes. The anti-BDNF, TrkB, p-CREB, CREB, p-ERK, and ERK antibodies (1:1000; Cell Signaling), and anti-p-TrkB (1:1000; Abcam), and anti-GAPDH (1:5000; Cell Signaling) were applied during an overnight incubation at 4 °C. Following this, membranes were incubated with appropriate secondary antibodies at room temperature (RT). The band intensities were visualized using the ECL Western Blotting Detection System (Amersham Biosciences, Pittsburgh, PA, USA) and analyzed using a luminescent image analyzer LAS-4000 (GE Healthcare, Uppsala, Sweden). Densitometric analysis of Western blotting data was performed using the Image J software (version 1.53) (NIH, Bethesda, MD, USA).

### 4.13. Quantitative Real-Time PCR (qRT-PCR)

Total RNA was extracted from the hippocampus of the mouse brain using Trizol reagent (Invitrogen, Waltham, MA, USA). The extracted RNA was preserved at −80 °C, and the concentration was determined using a NanoDrop spectrometer (ND-LITE, Thermo Fisher Scientific, Waltham, MA, USA). Of total RNA, 1 μg was synthesized into cDNA using amfiRivert cDNA Synthesis Platinum Master Mix (GenDEPOT, Katy, TX, USA). cDNA was used as a template for quantitative real-time PCR using amfiRivert qGreen Q-PCR master Mix (GenDEPOT). The primers used are as follows: mouse TNF-α forward: 5′-CCTGTAGCCCACGTCGTAGC-3′, reverse: 5′-TTGACCTCAGCGCTGAGTTG-3′; mouse IL-1β forward: 5′-GCTTTCAGGGGAGGGCT-3′, reverse: 5′-GTGCTCTGGTTGCTCTCTGT-3′; mouse BDNF forward: 5′-TGGCTGACATTTTGAGCACG-3′, reverse: 5′-GCTCCAAAGGCATTGACTGC-3′; rat GAPDH forward: 5′-CCATGGAGAAGGCTGGG-3′, reverse: 5′-CAAAGTTGTCATGGATGACC-3′. Gene expression was normalized to the mRNA levels of GAPDH.

### 4.14. Statistical Analysis

All data are expressed as mean ± standard error of the mean (SEM) and analyzed using GraphPad Prism 7 (GraphPad Software, Inc., La Jolla, CA, USA). The body weight was analyzed using two-way ANOVA with Tukey’s post hoc test and other numerical data were compared using Student’s t-test or one-way ANOVA with Dunnett’s post hoc test for unpaired observations between the two groups. For all analyses, statistical significance was set at *p* < 0.05.

## 5. Conclusions

These findings reveal that FPF protects mice from SD-induced depression-like and stress-coping behavior by regulating the HPA axis and the BDNF/TrkB/ERK/CREB signaling pathway. Although further study in which FPF is administrated as post-treatment after HPA axis dysfunction is induced by SD stress to evaluate the therapeutic efficacy of FPF, this study provides new insight into the therapeutic potential of FPF for SD-induced stress and depression.

## Data Availability

Not applicable.

## Supplementary Materials

The following supporting information can be downloaded at: https://www.mdpi.com/article/10.3390/ijms24010622/s1.
