# Supplementary figures and images for: Fermented Perilla frutescens Ameliorates Depression-like Behavior in Sleep-Deprivation-Induced Stress Model
Source: Int J Mol Sci. 2022 Dec 30;24(1):622. doi: 10.3390/ijms24010622 (PMC9820360; doi:10.3390/ijms24010622)

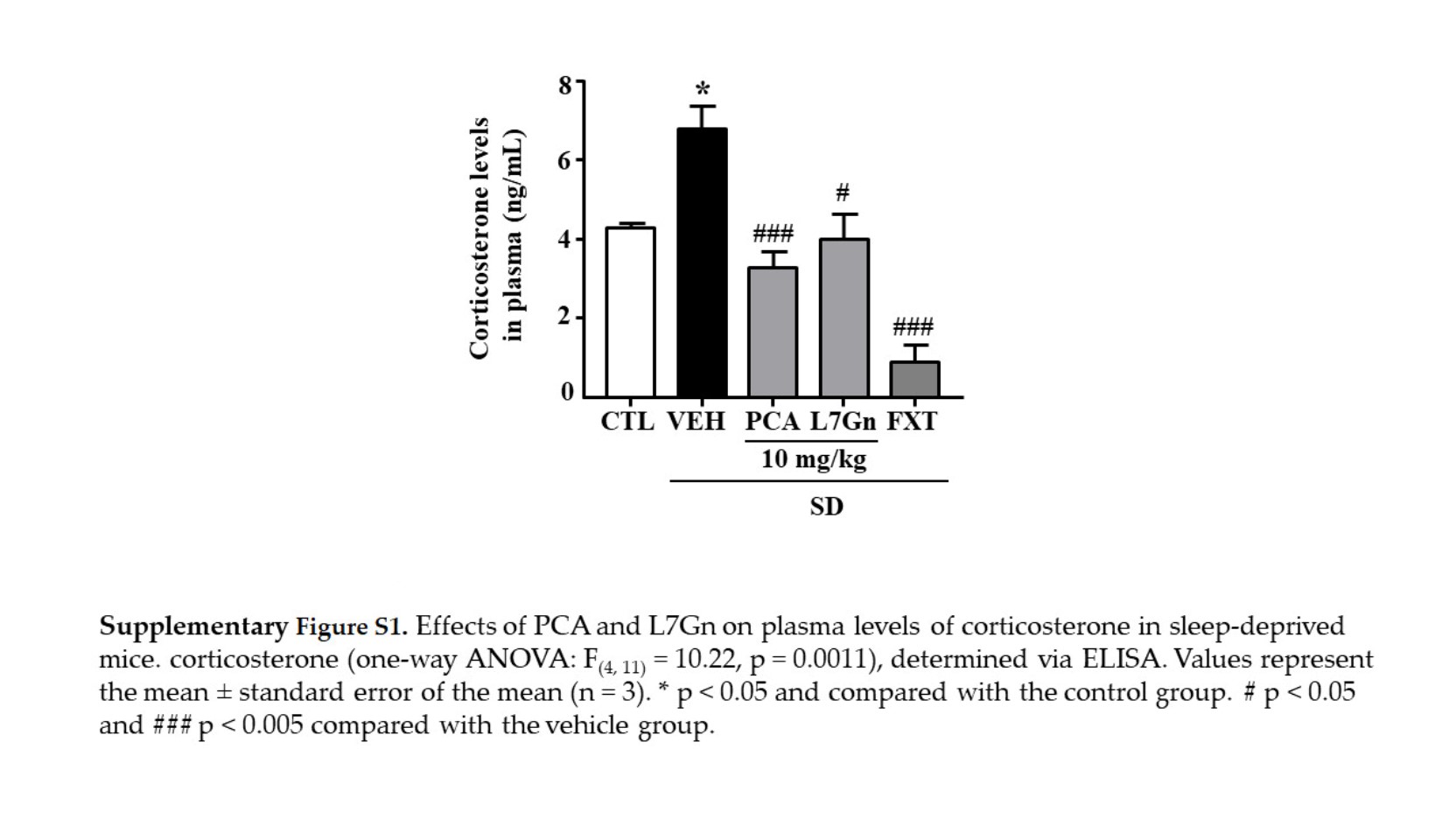

Supplement: Supplementary file 1 [file ijms-24-00622-s001.zip › ijms-2054301-supplementary.jpg]
